# Mitochondrial impairments in aetiopathology of multifactorial diseases: common origin but individual outcomes in context of 3P medicine

**DOI:** 10.1007/s13167-021-00237-2

**Published:** 2021-03-04

**Authors:** Lenka Koklesova, Marek Samec, Alena Liskova, Kevin Zhai, Dietrich Büsselberg, Frank A. Giordano, Peter Kubatka, Olga Golunitschaja

**Affiliations:** 1 Department of Obstetrics and Gynaecology, Jessenius Faculty of Medicine, Comenius University in Bratislava, 036 01 Martin, Slovakia; 2 Department of Physiology and Biophysics, Weill Cornell Medicine-Qatar, Education City, Qatar Foundation, Doha, 24144 Qatar; 3 Department of Radiation Oncology, University Hospital Bonn, Rheinische Friedrich-Wilhelms-Universität Bonn, 53127 Bonn, Germany; 4 Department of Medical Biology, Jessenius Faculty of Medicine, Comenius University in Bratislava, 03601 Martin, Slovakia; 5 Predictive, Preventive, Personalised (3P) Medicine, Department of Radiation Oncology, University Hospital Bonn, Rheinische Friedrich-Wilhelms-Universität Bonn, 53127 Bonn, Germany

**Keywords:** Mitochondrial impairment, Injury, ROS, Energy imbalance, Vicious circle, Oxidative stress, Aetiopathology multi-organ dysfunction, Suboptimal health, Reversible damage, Vasospasm, Mitigating measures, Repair, Mechanisms, Multifactorial disease, Ageing, Neurodegeneration, Glaucoma, Alzheimer, Cancer, Cardiovascular disease, Predictive preventive personalised medicine (PPPM/3PM), Multi-modal diagnostics, Liquid biopsy, Molecular patterns, Biomarker panel, Patient stratification, Individualised patient profiling, Origin, Outcomes, Complementary medicine, Health policy, Cost efficacy, COVID-19

## Abstract

Mitochondrial injury plays a key role in the aetiopathology of multifactorial diseases exhibiting a “vicious circle” characteristic for pathomechanisms of the mitochondrial and multi-organ damage frequently developed in a reciprocal manner. Although the origin of the damage is common (uncontrolled ROS release, diminished energy production and extensive oxidative stress to life-important biomolecules such as mtDNA and chrDNA), individual outcomes differ significantly representing a spectrum of associated pathologies including but not restricted to neurodegeneration, cardiovascular diseases and cancers. Contextually, the role of predictive, preventive and personalised (PPPM/3P) medicine is to introduce predictive analytical approaches which allow for distinguishing between individual outcomes under circumstance of mitochondrial impairments followed by cost-effective targeted prevention and personalisation of medical services. Current article considers innovative concepts and analytical instruments to advance management of mitochondriopathies and associated pathologies.

## Introduction

### “Vicious circle” of the mitochondrial injury and multi-organ dysfunction

Mitochondria are semi-autonomous organelles of prokaryotic origin [1], with outer and inner membranes encapsulating the intermembrane space and matrix compartments [2]. Proper mitochondrial physiology is essential for maintaining physical and mental health. Mitochondria primarily act as the main energy supplier through oxidative phosphorylation (OXPHOS) and therefore directly influencing the efficacy of highly energy-consuming repair process in the cell [3]. Mitochondria are involved in regulation of ion homeostasis, redox potential, lipid metabolism, metabolite synthesis, cell differentiation, immune system as well as anti-apoptotic and anti-ageing processes, amongst others [4–9].

The mitochondrial genome is represented by 16,569 bp mitochondrial DNA (mtDNA). In animals and humans, inheritance of mtDNA is considered to be exclusively of maternal origin [10]. Accumulation of mtDNA mutations is associated with accelerated ageing and development of ageing-associated pathologies such as neurological disorders, cardiovascular diseases (CVDs), metabolic syndromes and cancers [3, 10]. Mitochondrial impairments (known also as mitochondriopathies) can be inherited (through an autosome and/or X chromosome maternally) or developed in a multi-factorial way including but not restricted to a toxic environment, sub-optimal health conditions and collateral pathologies (such as metabolic syndrome) [3]. Mitochondriopathies carry systemic character and can be damaging for many organs [11]. Molecular interplay shifted towards excessive ROS formation, but diminished energy production is a critical “vicious circle” of the mitochondrial injury and multi-organ dysfunction which can be developed in a reciprocal manner [3]. By insufficient energy production, chronic exposure to ROS overproduction consequently leads to the oxidative damage of life-important biomolecules including nucleic acids, proteins, lipids and amino acids, amongst others. Consequently, mitochondrial dysfunction is associated with accelerated ageing, neurodegeneration, tumourigenesis, metabolic syndromes and mood disorders, amongst others [3]. As the multi-factorial disorder of different severity grade, mitochondriopathies are remarkably heterogeneous being, therefore, challenging for overall clinical management.

Regarding diagnostics, since different forms of mitochondrial dysfunction may affect the brain, heart, peripheral nervous and endocrine systems, eyes, ears, guts and kidney, amongst other organs, mitochondriopathies have been proposed as an attractive diagnostic target to be investigated in any patient with unexplained progressive multisystem disorder [3].

Approaches to treat neurodegenerative disorders such as Alzheimer’s disease and glaucoma include standardised ginkgo biloba extract (EGb761®), piracetam and Dimebon, which are known to address many aspects of mitochondrial functionality such as mitochondrial dynamics [11]. Further, generalised approaches such as physical exercise demonstrating neuroprotective [12], cardioprotective [13–18] and anti-cancer [19] effects are clearly associated with the mitochondrial function support. Finally, phytochemicals, naturally occurring compounds, used due to their neuroprotective, cardioprotective and anti-carcinogenic effects, have been demonstrated as a modulator of the mitochondrial function, structure and related mechanisms [20–27].

### Mitochondriopathies in focus of predictive approach, targeted prevention and personalisation of medical services

Mitochondriopathies, concomitant multi-organ damage and associated broad spectrum of chronic disorders cause enormous socio-economic burden. Contextually, the paradigm change from reactive medicine to PPPM strategies is strongly recommended to advance healthcare in the area [28].

Due to absent causative therapies and cure for individual forms of mitochondriopathies, predictive approaches, individualised patient profiling, targeted prevention and personalisation of medical services are instrumental for the overall management of mitochondriopathies.

This article details pathomechanisms related to mitochondrial injury as the clue to multi-factorial disorders and exemplifies conditions and tools to be considered at the clinical side, in order to identify predisposed individuals and to introduce targeted mitigating measures against potential mitochondriopathy and cascaded development of related pathologies.

## Multifunctionality of mitochondria in maintaining physical and mental health versus disease development

**Fig. 1:**
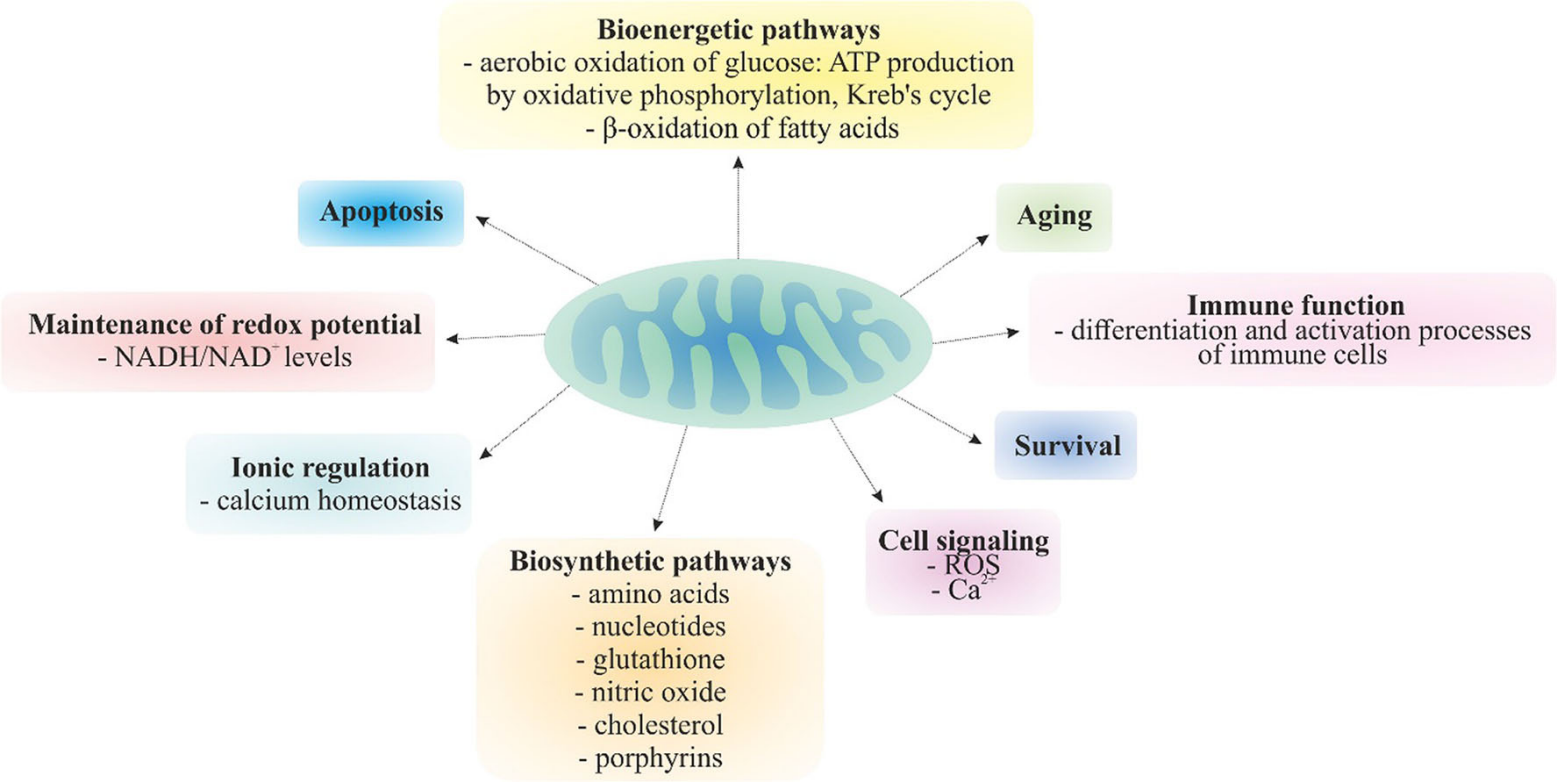
Mitochondrial function in the cell

Mitochondria perform an essential role in eukaryotic organisms with important cellular functions, especially in energy metabolism, and also in synthetic and oxidation/reduction processes, ionic regulation (e.g. calcium homeostasis) and signalling pathways connected to cell communication, survival and death [4–7, 10]. The key role of mitochondria in the physiology of cells needed for maintaining physical and mental health is summarised in Fig. 1. To this end, as described for a broad spectrum of cell types, mitochondria are highly heterogeneous considering their morphology and functionality that should be kept in mind considering tissue and organ specificity [29].

**Fig. 2:**
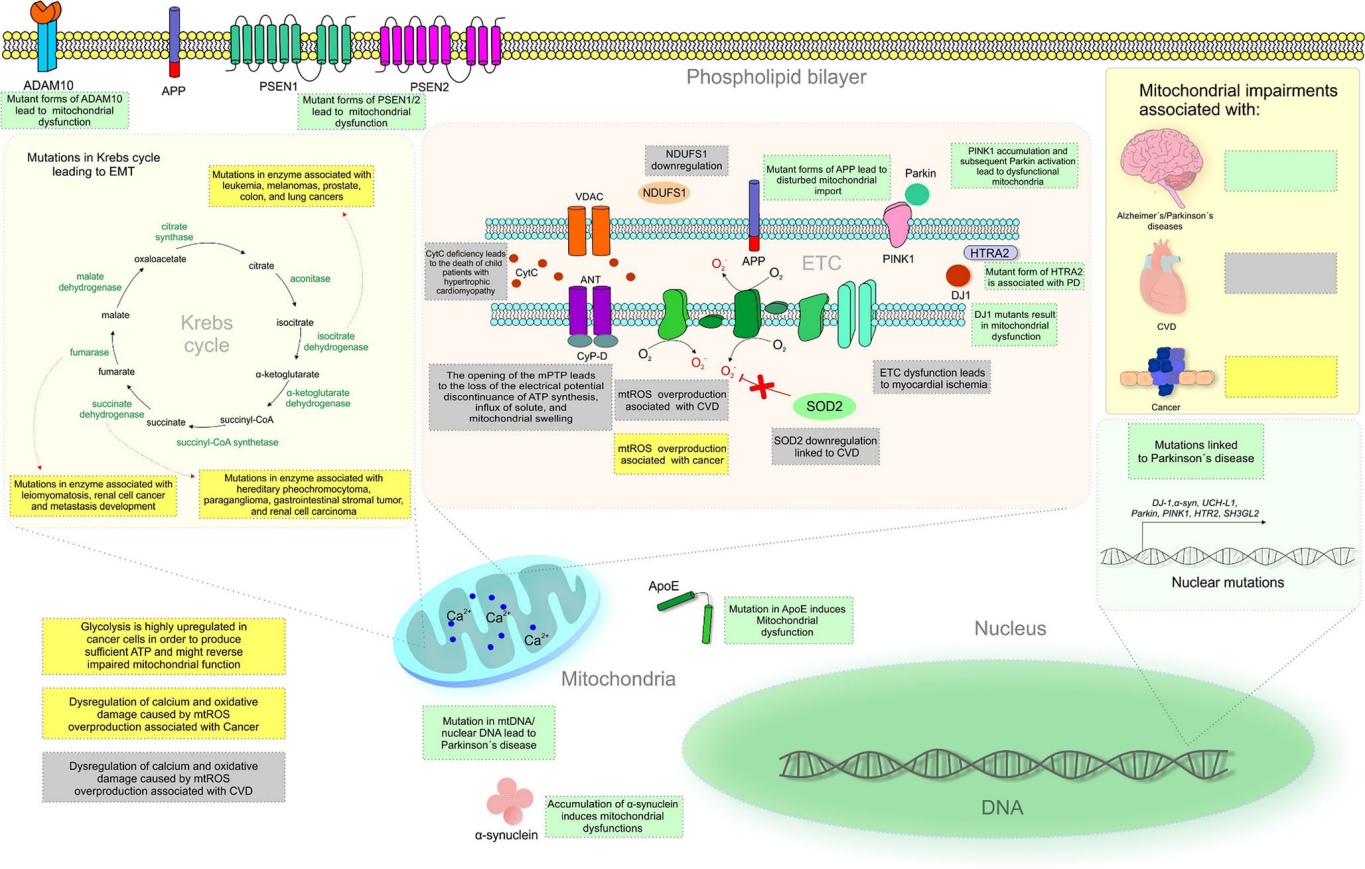
Molecular mechanisms and factors associated with mitochondrial impairments in neurodegenerative and cardiovascular diseases, and cancer. **A**DAM10, a disintegrin and 71 metalloprotease 10; APP, amyloid precursor protein; PSEN1, presenilin 1; PSEN2, presenilin 2; EMT, epithelial-mesenchymal transition; mtROS, mitochondrial reactive oxygen species; CVD, cardiovascular disease; DNA, deoxyribonucleic acid; ApoE, apolipoprotein E; SOD2, superoxide dismutase 2; ETC, electron transport chain; DJ1, parkin-associated protein involved with oxidative stress; HTRA2, serine peptidase 2; PD, Parkinson’s disease; mPTP, mitochondrial permeability transition pore; ATP, adenosine triphosphate; PINK1, putative serine threonine kinase; CytC, cytochrome *c*; ANT, adenine nucleotide translocator; CyPD, cyclophilin D; NDUFS1, anti-oxidative enzyme superoxide dismutase 2 and complex I subunit; VDAC, voltage-dependent anion channel; Parkin, E3 ubiquitin ligase; α-syn, α-synuclein; UCH-L1, ubiquitin carboxy-terminal hydrolase L1; SH3GL2, SH3 domain containing GRB2 like 2/endophilin A1

Human mitochondria contain 16,569 bp circular DNA which encodes 37 genes for ribonucleic acids (RNAs) and protein subunits of the respiratory chain [30]. Noteworthy, mtDNA usually demonstrates higher mutation rates compared with these of chromosomal DNA; accumulation of mtDNA mutations has been related to ageing and age-associated diseases [31]. Being the major producers of reactive oxygen species (ROS) in the cell, mitochondria are extensively exposed to the oxidative damage [32]. However, under physiologic condition, controlled production of ROS, sufficient energy supply and efficient repair performance are well-balanced together [3]. Under this condition, mtDNA, damaged by oxidative stress, can be effectively repaired through base excision repair (BER) to restore mitochondrial genome integrity. In contrast, under the “vicious circle” circumstances, uncontrolled ROS overproduction accompanied with diminished energy supply and repair machinery insufficiency collectively results in extensive mutations within the mtDNA including genes responsible for the BER pathway and mitochondrial repair enzymatic activities; irreversible changes in mitochondrial dynamics, including mitochondrial fusion/fission, motility, morphology, size and transport [33]; and irreversible damage to life-important biomolecules and development of associated diseases [3]. Figure 2 presents molecular mechanisms and factors responsible for mitochondrial impairments associated with a spectrum of neurodegenerative disabilities, CVDs and cancers.

Declined mitochondrial qualities and activities are associated with multi-faceted ageing processes; in turn, ageing-related accumulation of mitochondrial mutations predisposes affected individual to a wide range of related disorders such as metabolic syndromes, cancers, CVDs and multiple neurodegeneration [34]. Mitochondrial ageing observed in associated disorders is characterised by the reciprocal relationship between the decreased respiratory capacity and uncontrolled ROS overproduction leading to strongly pronounced oxidative stress, increased pyruvate oxidation, telomere shortening, lipid toxicity and metabolic disturbances such as reduced both an activity of citrate synthase and phosphocreatine recovery time and, finally, dysfunctional mitochondrial quality control machinery (seen, e.g. in autophagy) [35, 36].

## Mitochondrial impairments characteristic for neurodegeneration

Progressive neurodegeneration causes neuronal death and synapse loss in vulnerable areas of the spinal cord and brain as well as visual impairments and blindness by retinal diseases. Regarding the latter, oxidative stress and associated mitochondrial dysfunction are integral components of the aetiopathology of retinal diseases, including diabetic retinopathy, age-related macular degeneration and glaucoma [37]. Both non-modifiable and modifiable (preventable) multi-factorial risk factors are involved in neurodegenerative process including by not restricted to the genetic predisposition, suboptimal health conditions, toxic environment, endogenous and exogenous stress, systemic ischemia-reperfusion and mitochondrial vulnerability, which individually or collectively may lead to irreversible damage and degeneration of neuronal systems [38–40].

In glaucomatous optic nerve degeneration considered the second leading cause of blindness in human beings, currently affecting around 70 million patients worldwide, an oxidative stress by ischemia-reperfusion linked to mitochondrial impairments, insufficient DNA repair and neuronal damage, amongst other related processes has been demonstrated as belonging to the comprehensive aetiopathology of the disease [41, 42]. Further, as detailed specifically for the normal-tension glaucoma, an imbalanced vasoconstriction in response to multi-factorial stimuli (such as cold provocation, hormonal and emotional stress) plays a central role in systemic ischemia-reperfusion damage and can be observed early in life of persons with suboptimal health conditions such as vasospastic individuals [43]. Consequently, the subpopulation of young vasospastic individuals demonstrating reversible systemic damages is considered as a potent target for innovative screening programmes and application of predictive diagnosis, cost-effective targeted prevention and treatment algorithms tailored to the personalised patient profiles [44].

Alzheimer’s disease is one of the most prominent examples of a multi-factorial neurodegenerative disorder related to oxidative stress and mitochondrial dysfunction with high morbidity and mortality registered worldwide [45]. At early pathological events, synaptic damage correlates strongly with cognitive deficits and memory loss. Alzheimer’s disease is related to the increased production and impaired clearance of self-aggregating forms of β-amyloid [46]. Moreover, mtDNA frequently encodes ETC components; therefore, mtDNA mutations lead to increased mitochondrial energetic dysfunction [47]. Besides, various gene mutations closely associated with mitochondrial function, including those involving amyloid precursor protein (*APP*), presenilin 1 (*PSEN1*), 46 presenilin 2 (*PSEN2*), apolipoprotein E (*ApoE*) and a disintegrin and 71 metalloprotease 10 (*ADAM10*), lead to Alzheimer’s disease [48]. APP accumulated in mitochondrial protein import channels interacts with various mitochondrial proteins and leads to mitochondrial dysfunction [49]. Moreover, neuronal damage or stress leads to ApoE synthesis. In neurons, the specific conformation of ApoE4 is susceptible to proteolysis, resulting in pathological mitochondrial dysfunction and cytoskeletal alterations [50]. PSEN mutations enhance neurodegeneration mediated by endoplasmic reticulum-mitochondria calcium transfer [51]. Finally, mutations in ADAM10 increase β-amyloid production and mitochondrial impairment associated with Alzheimer’s disease pathogenesis [52].

Parkinson’s disease affects about 2% of the population above the age of 60 years [53]. Parkinson’s disease is characterised by the loss of dopaminergic neurons in the substantia nigra pars compacta (SNpc) and the presence of misfolded α-synuclein (α-syn) in intra-cytoplasmic inclusions known as Lewy bodies [54]. Mutations in mtDNA or nuclear DNA, including those involving E3 ubiquitin ligase (*Parkin)*, α-syn, ubiquitin carboxy-terminal hydrolase L1 (*UCHL1*), parkin-associated protein involved with oxidative stress (*DJ1*), putative serine threonine kinase (*PINK1*), auxilin (*DNAJC6*), synaptojanin 1 (*SYNJ1*), serine peptidase 2 (*HTRA2)* and endophilin A1 (*SH3GL2*), are described in the pathogenesis of Parkinson’s disease [55–59]. These genes are important for mitochondrial function, and mutations or disturbances in function can lead to mitochondrial impairments. *α-syn* controls mitochondrial function under both physiological and pathological conditions. Mutations in *α-syn* contribute to neuronal impairment in Parkinson’s disease [60]. *DJ1* encodes a ubiquitous, highly conserved protein. DJ1 is an integral mitochondrial protein that maintains the activity of mitochondrial complex I and regulates mitochondrial homeostasis [61]. Moreover, the accumulation of PINK1 on defective mitochondria leads to the translocation of Parkin from the cytosol to eliminate damaged mitochondria through mitophagy (the selective degradation of mitochondria by autophagy) [62]. Disruptions in mitochondrial homeostasis or the expression of PINK1 and Parkin leads to mitochondrial impairments and associated disorders such as Parkinson’s disease. Moreover, HTRA2 is a mitochondrial protein with a proteolytic role in protein quality control and homeostasis in the mitochondrial intermembrane space. Mutations in HTRA2 are associated with autosomal dominant late-onset Parkinson’s disease [63]. Furthermore, UCHL1 is a key enzyme in the protein degradation pathway and functions in the physiological remodelling of synapses by controlling ubiquitin homeostasis. Any disturbance in homeostasis contributes to mitochondrial and synaptic failure [64]. Finally, *DNAJC6*, *SYNJ1* and *SH3GL2* are associated with the disruption of synaptic vesicle endocytosis, which contributes to mitochondrial dysfunction and is thus related to the pathogenesis of Parkinson’s disease.

## Mitochondrial impairments characteristic for cardiovascular diseases

CVDs, a prevalent cause of morbidity and mortality worldwide, comprise heart and circulatory system disorders which result mainly from atherosclerosis and manifest as heart attacks and strokes [65, 66]. CVDs are highly heterogeneous and chronic diseases which may remain asymptomatic for a long time [67]. Several factors are responsible for the development of CVDs including invariable factors, such as gender, age and genetic heritage, versus variable factors, such as sedentary life-style, tobacco use, obesity, inappropriate eating habits, high blood pressure and preventable metabolic syndromes, amongst others [68]. Moreover, there are some gender specific risk factors such as related to female hormonal regulation in peri/menopause and pregnancy [69].

Mitochondria play an important role in cardiac homeostasis. Being highly energy-consuming, cardiomyocytes are rich on mitochondria. Deficient ATP synthesis and energy metabolism contribute in a reciprocal way to disturbed cardiac excitation-contraction, severe mitochondrial impairments and development of CVDs, including atherosclerosis, ischemic heart disease, cardiac hypertrophy and heart failure [70–72]. Mitochondrial impairments associated with CVDs are characterised by enhanced ROS production, intracellular ATP depletion, extensive cell damage and highly increased cardiomyocyte apoptotic rates [70]. Stress conditions can lead to calcium and ROS overload, resulting in the loss of mitochondrial membrane potential and the consequent release of mitochondrial proteins including cytochrome *c* (CytC) [73, 74]. CytC deficiency in children diagnosed with hypertrophic cardiomyopathy is a known mortality cause [75]. Noteworthy, accumulating mtDNA mutations have been associated with ischemic heart disease, cardiomyopathy, atherosclerotic vascular disease, dysrhythmias and heart failure [76].

Ischemic heart disease, also known as coronary heart disease, is characterised by an inadequate blood supply to the heart caused by the blockage of blood vessels [77]. To this end, insufficient coronary micro-vessel dilatation, coronary microvascular spasms and dysfunction and extravascular compressive forces contribute to chronic and acute forms of ischemic heart disease [77]. Furthermore, damage in the ETC is responsible for severe myocardial ischemia [78]. In stressed cells, mitochondria activate death channels, especially the mitochondrial permeability transition pore (mPTP), which is regulated by several proteins, including the voltage-dependent anion channel (VDAC), the adenine nucleotide translocator (ANT) and cyclophilin D (CypD). The opening of the mPTP immediately disrupts the electrical potential, halting ATP synthesis and causing an influx of solute and mitochondrial swelling [79–81].

ATP depletion is heavily implicated in both ischemic heart disease and heart failure. Heart failure represents a complex clinical syndrome associated with impaired contractile performance of the myocardium and the heart’s inability to sufficiently perfuse peripheral tissues [70]. A major reason for heart failure is calcium dysregulation and oxidative damage caused by mtROS overproduction in human patients [82, 83]. Moreover, significant mtDNA depletion and inhibition of the expression of mtDNA-encoded proteins are observed in the human heart failure [84]. Cardiac metabolism in the pathological state exhibits an increased reliance on glucose and therefore glycolysis [85]. Systolic heart failure can be further associated with hypertension and/or diabetes [86]. Patients with combined chronic heart failure and diabetes mellitus have worse prognoses associated with elevated ROS overproduction but decreased SOD2/NDUFS1 expression rates compared to patients with chronic heart failure without diabetic history [87].

## Mitochondrial impairments characteristic for cancers

In malignancies, metastasis is the main cause of death in more than 90% of cancer patients [88]. The heterogeneity of cancers and their frequent therapeutic resistance [89, 90] are further concerns motivating application of innovative cost-effective approaches by predictive diagnostics of reversible damage, risk assessment, targeted prevention and treatment algorithms tailored to the person [19, 91–96] whereas about 5 to 10% of cancers are caused by inherited predisposition to malignant cell transformation [97]; the majority of cancer cases carry a sporadic character being preventable [19, 93, 98].

Accumulating mtDNA mutations and uncontrolled ROS overproduction are characteristic for solid and haematological malignancies [99–103] both associated with genomic instability and irreversible alterations in gene expression patterns and related signalling pathways. Concomitant changes in Ca^2+^ and onco-metabolite concentrations are highly relevant for mitochondrial retrograde signalling, neoplastic transformation and cancer progression [104]. Disturbed homeostasis of mitochondrial energy metabolism is crucial for the malignant cell transformation and metastatic disease known as the Warburg effect and characterised by the switch from OXPHOS to glycolysis [93]*.* To this end, activation of hypoxia-inducible factor 1 (HIF-1) by oncogenic protein kinase B (AKT) suppresses pyruvate dehydrogenase (PDH) activity [105]. Upregulation of glucose 6-phosphate dehydrogenase, pyruvate kinase M2 and Rad6 and downregulation of succinate dehydrogenase further contribute to higher lactate levels associated with the Warburg effect [106].

Mutations occur to the mitochondrial Krebs cycle genes contribute to tumorigenesis through the epithelial-mesenchymal transition (EMT). Mitochondrial dysfunction (i.e. OXPHOS downregulation) promotes EMT increasing cancer aggressiveness and poor individual outcomes [107]. Mutations in fumarate hydratase inhibit the conversion of fumarate to malate and lead to leiomyomatosis and highly aggressive renal cell cancer with early-stage metastasis [108]. Mutations of the isocitrate dehydrogenase promote oxidative decarboxylation of isocitrate to α-ketoglutarate demonstrated for several cancer types including leukaemia, melanoma, and prostate, colon and lung cancers [109]. Mutations in succinate dehydrogenase predispose to pheochromocytoma, paraganglioma and gastrointestinal stromal tumours as well as renal cell carcinoma [110].

## Common origin but individual outcomes

As detailed above, mitochondrial injury and consequently disturbed energy homeostasis and uncontrolled ROS overproduction cause/strongly contribute to neurodegeneration, malignant cell transformation and CVDs. Moreover, a number of disrupted mitochondrial genes are overlapped in the development of all these pathologies. For example, *Parkin* associated with Parkinson’s disease acts also in hepatocellular carcinoma [111]. Aberrantly expressed and methylated α-syn, on one hand, can contribute to neuronal impairment in Parkinson’s disease [60] and, on the other hand, has been found in different cancer types including melanoma and brain, ovarian, breast and colorectal cancers [112, 113].

Upregulation of phosphorylated microtubule-associated protein tau (*MAPT*) and consequently altered mitochondrial functions are associated with Alzheimer’s disease [114]; in cancer, *MAPT* overexpression is linked to poor prognosis and drug resistance [115]. Mitochondrial impairments, especially dysfunction of respiratory complex II, cause excessive mtROS generation that is known to be involved in the pathogenesis of both—development of familiar and sporadic cancers and neurodegenerative disorders [116]. The paradox is that although the disease origin is common, individually outcomes differ from each other. Below we provide recently collected statistics demonstrating that, for example, neurodegenerative processes seem to protect against cancer development.

Smoking- and non-smoking-associated cancers occur less frequently in patients with Parkinson’s disease [117–119]*.* No associations between Parkinson’s disease and nonfatal cancers were observed [120]. A meta-analysis of fifteen studies comprising 346,153 Parkinson’s disease cases demonstrated a lower risk of prostate cancer in the Western population [121]. Although Alzheimer’s disease and cancer share multiple impairments related to the ATP depletion, mitochondrial injury and decreased PDH activity [121], patients with Alzheimer’s disease demonstrate lower cancer risk compared to the general population [122–124]. A large Danish nationwide cohort study revealed inverse associations between Alzheimer’s disease and subsequent cancer diagnoses, specifically pronounced for breast cancer and melanoma compared to the general population [125]. No association have been demonstrated between Parkinson’s disease and risks for several cancers including breast, digestive system, lung, urinary and reproductive system cancers as well as haematological malignancies [126]. Further statistics demonstrate that cancer patients are at greater risk to develop later on Parkinson’s or Alzheimer’s disease, in contrast to lower risk to disease on cancer for people affected by neurodegeneration [127]. This phenomenon hypothetically might be explained by side effects of cancer treatments similarly to a highly increased ischemic stroke incidence well-known for patients with the cancer treatment history: for almost all cancers survivors, the risk of stroke increases with time [128].

In summary, neurodegeneration, cancers and CVDs share many common risk factors and molecular pathways related to mitochondrial function and impairments [129–131]. However, still individual areas undergo rather separate investigations that limits their analytical power and create barriers in development of personalised predictive diagnostics and application of cost-effective targeted prevention. Consequently, it is strongly recommended to reconsider future analytical strategies in favour of more comprehensive approaches aiming at multi-modal diagnostics which allow for prediction of individual outcomes under circumstances of mitochondrial impairments [3].

## Liquid biopsy is instrumental for individualised diagnostics and prediction of pathologies associated with mitochondriopathies

Table 1 summarises the research focusing on potential biomarkers obtained from liquid biopsy that are important for improved individualised diagnostics and prediction of pathologies associated with mitochondriopathies, especially neurodegenerative disorders, CVDs and cancer.

**Table 1:** Liquid biopsy in individualised diagnostics and prediction of pathologies associated with mitochondriopathies

Biomarker	Type of mitochondrial disease	Fluid sample	Results	Reference
mtDNA	Alzheimer’s disease patients	Cerebrospinal fluid	↓ mtDNA in presymptomatic patients with *PSEN1* mutation	[132]
ApoE			↑ ApoE compared to control	[133]
Oxidant and antioxidant metabolites		Blood	↑ Oxidative stress, ↑ hydrogen peroxide, ↑ organic hydroperoxides, ↓ GSH/GSSG ratio, ↓ GSH transferase, ↓ ATP compared to young adult control	[134]
Lipofuscin-like pigments			↑ Lipofuscin-like pigments compared to control	[135]
β-amyloid		Plasma	↑ β-amyloid in Alzheimer’s disease patients, ↓ β-amyloid after vitamin D treatment, ↓ β-amyloid-related biomarkers (Aβ42, APP, BACE1, APPmRNA, BACE1mRNA)	[136]
8-OHdG		Urine	Different levels of 8-OHdG and 2’-deoxyguanosine between patients with Alzheimer’s disease and healthy control	[137]
AD7C neural thread protein			↑ AD7C neural thread protein in Alzheimer’s disease patients compared to non- Alzheimer’s disease dementia, and healthy normal individuals	[138]
DJ1	Parkinson’s disease patients	Cerebrospinal fluid	↑ DJ1 compared to control	[139]
Advanced oxidised protein products		Cerebrospinal fluid and serum	↑ Advanced oxidised protein products compared to control	[140]
ROS and SOD		Blood	↑ mtROS in monocytes, ↓ antioxidant SOD in blood	[141]
Oxidative stress markers			↓ GSH peroxidase, ↑ oxidised GSH, ↑ MDA contents	[142]
Uric acid		Serum	↓ Uric acid	[143]
Biopyrrin		Urine	↑ Biopyrrin compared to control	[144]
ApoC3	Coronary heart disease patients	Serum	↓ ApoC3, ↓ triglyceride after aerobic exercise for 8 weeks compared to baseline	[145]
Cardiac troponin I	Patients with acute decompensated heart failure		↑ Cardiac troponin I is associated with poor prognosis and increased mortality	[146]
N-terminal portion of pro-brain natriuretic peptide and adrenomedullin	Ischemic heart disease patients	Plasma	↑ N-terminal portion of pro-brain natriuretic peptide and adrenomedullin predict heart failure and death	[147]
Tumour necrosis factor-α receptor-1 and brain natriuretic peptide	Ischemic heart failure patients		Levels are highly predictive for the primary end point of death or cardiac hospitalisation	[148]
D-dimer	Stroke and coronary heart disease patients		↑ Basal plasma level of d-dimer is associated with ischemic stroke, especially cardioembolic stroke	[149]
Hsa_circ_0001445	Coronary artery disease patients	Plasma	Levels of hsa_circ_0001445 are proportional to coronary atherosclerotic burden	[150]
Hsa_circ_0001879 and hsa_circ_0004104		Blood	↑ hsa_circ_0001879 and hsa_circ_0004104 compared to control	[151]
8OHdG	Lung cancer patients	Blood	↑ 8OHdG compared to healthy control	[152]
	Prostate cancer patients		↑ 8OHdG in high-risk patients	[153]
MDA, GSSG, GSH, TAC	Breast cancer patients		↑ MDA, ↑ GSSG, ↓ GSH, ↓ TAC, ↓ GSH/GSSG ratio compared to control	[154]
Diacron’s reactive oxygen metabolites and total thiol level	Colorectal cancer patients		↑ Diacron’s reactive oxygen metabolites, ↓ total thiol level	[155]
MtDNA copy number	Acute lymphoblastic leukaemia patients		↑ mtDNA copy number, ↑ mitochondrial deletion ratios, ↓ mtDNA copy number after chemotherapy compared to controls	[156]
TOM34 and HSP90AA1	Hepatocellular carcinoma patients	Serum	↑ TOM34, ↑ HSP90AA1 compared to cirrhotic patients	[157]

↑ Increased

↓ Decreased

Abbreviation: *mtDNA* mitochondrial DNA, *PSEN1* presenilin 1, *ApoE* apolipoprotein E, *GSH* glutathione, *GSSG* glutathione disulphide, *ATP* adenosine triphosphate, *Aβ42* the 42 amino acid form of amyloid-β, *APP* amyloid-β precursor protein, *BACE1* β-secretase 1, *DJ1* parkin-associated protein involved with oxidative stress, *ROS* reactive oxygen species, *SOD* superoxide dismutase, *MDA* malondialdehyde, *ApoC3* apolipoprotein C3, *8OHdG* 8-hydroxy-2′–deoxyguanosine, *TAC* total antioxidant capacity, *TOM34* 34-kDa translocase of the outer mitochondrial membrane, *HSP90AA1* heat shock protein 90 alpha family class A member 1

## Conclusions and expert recommendations

Mitochondrial injury plays a key role in the aetiopathology of multifactorial diseases exhibiting a “vicious circle” characteristic for the mitochondrial and multi-organ damage frequently developed in a reciprocal manner. Although the origin is common (uncontrolled ROS release, diminished energy production and extensive oxidative stress to life-important biomolecules such as mtDNA and chrDNA), individual outcomes differ significantly from each other comprising a spectrum of associated pathologies including but not restricted to the neurodegeneration, CVDs and cancers. Although corresponding pathomechanisms and molecular pathways overlap between individual mitochondriopathy-related pathologies, multi-centre studies demonstrate that, for example, neurodegenerative processes seem to protect against cancer development. In contrast, cancer patients are at greater risk to develop later on Parkinson’s or Alzheimer’s disease—the phenomenon which hypothetically might be explained by side effects of cancer treatments similarly to a highly increased ischemic stroke incidence well-known for patients with the cancer treatment history. Unfortunately, individual areas currently undergo rather separate investigations that limit their analytical power and create barriers in development of personalised predictive diagnostics and application of cost-effective targeted prevention.

Contextually, the role of predictive, preventive and personalised (PPPM/3P) medicine is to force innovative analytical approaches which would allow for distinguishing between individual outcomes under circumstance of mitochondrial impairments. For that, individualised patient profiling, patient stratification, screening programmes focused on suboptimal health conditions, non-invasive prediction by applying liquid biopsies and cost-effective targeted prevention are instrumental for the paradigm shift from reactive medicine to PPPM. Recent progress made in the area of mitochondriopathies revealed that patient stratification and risk assessment are supportive for the effective treatments considering the level of mitochondrial impairment and individual predisposition to associated pathologies [158, 159]. General mitigating measures against oxidative damage include application of antioxidant agents with scavenging activity such as phytochemicals [21, 94, 160], personally adapted physical activities, dietary habits and individualised life-style recommendations [19]. Further, individualised phenotyping is instrumental for screening programmes focused on individuals with reversible damage such as young vasospastic individuals with systemic ischemic-reperfusion effects clearly predisposed to mitochondrial injury and associated pathologies [40, 44, 91].

Last but not the least, acute pandemic conditions require effective predictive, preventive and personalised algorithms for correct decisions made at clinical side. Viral infections are known to provoke necrosis, which amplifies anti-viral immune responses releasing damage-associated molecular patterns. Severely affected cells and tissues intrinsically secrete cell-free nucleic acids such as mtDNA. Indeed, COVID-19 patients with increased mtDNA levels are at elevated death risk and have to be intubated. Consequently, cell-free mtDNA is a potential biomarker for individualised survival status prediction in COVID-19 patients as a model for predictive approach under pandemic conditions [38, 161, 162].

## Data Availability

Not applicable.

## Abbreviations

ROS: Reactive oxygen species
mtROS: Mitochondrial reactive oxygen species
PPPM/3PM: Predictive preventive personalised medicine
mtDNA: Mitochondrial DNA
chrDNA: Chromosomal DNA
OXPHO: Oxidative phosphorylation
CVDs: Cardiovascular diseases
BER: Base excision repair
ADAM1: A disintegrin and 71 metalloprotease 10
APP: Amyloid precursor protein
PSEN1: Presenilin 1
PSEN2: Presenilin 2
EMT: Epithelial-mesenchymal transition
Apo: Apolipoprotein
SOD: Superoxide dismutase
ETC: Electron transport chain
DJ1: Parkin-associated protein involved with oxidative stress
HTRA2: Serine peptidase 2
mPTP: Mitochondrial permeability transition pore
ATP: Adenosine triphosphate
PINK1: Putative serine threonine kinase
CytC: Cytochrome *c*
ANT: Adenine nucleotide translocator
CypD: Cyclophilin D
ANT: Adenine nucleotide translocator
NDUFS1: Anti-oxidative enzyme superoxide dismutase 2 and complex I subunit
VDAC: Voltage-dependent anion channel
Parkin: E3 ubiquitin ligase
α-syn: α-Synuclein
UCHL1: Ubiquitin carboxy-terminal hydrolase L1
SH3GL2: SH3 domain containing GRB2 like 2/endophilin A1
DNAJC6: Auxilin
SYNJ1: Synaptojanin 1
SNpc: Substantia nigra pars compacta
Ca^2+^: Calcium cation
HIF-1: Hypoxia-inducible factor 1
AKT: Oncogenic protein kinase B
PDH: Pyruvate dehydrogenase
MAPT: Microtubule-associated protein tau
GSH: Glutathione
GSSG: Glutathione disulphide
Aβ42: The 42 amino acid form of amyloid-β
BACE1: β-secretase 1
MDA: Malondialdehyde
8OHdG: 8-Hydroxy-2′–deoxyguanosine
TAC: Total antioxidant capacity
TOM34: 34-kDa translocase of the outer mitochondrial membrane
HSP90AA1: Heat shock protein 90 alpha family class A member 1
